# Oral Behaviors, Anxiety, and Depression in Temporomandibular Disorders: A Conceptual Narrative Review Within the DC/TMD Axis II Framework

**DOI:** 10.3390/medicina62050999

**Published:** 2026-05-20

**Authors:** Alexandra Lavinia Vlad, Ioana Scrobota, Raluca Ortensia Cristina Iurcov, Ioan Andrei Țig, Anca Maria Fratila, Gabriela Ciavoi

**Affiliations:** 1 Doctoral School of Biomedical Sciences, Faculty of Medicine and Pharmacy, University of Oradea, 410087 Oradea, Romania; alvlad@uoradea.ro (A.L.V.); gciavoi@uoradea.ro (G.C.); 2 Department of Dental Medicine, Faculty of Medicine and Pharmacy, University of Oradea, 410068 Oradea, Romania; itig@uoradea.ro; 3 Faculty of Medicine, Lucian Blaga University of Sibiu, 550169 Sibiu, Romania; anca.fratila@ulbsibiu.ro; 4 Military Clinical Emergency Hospital of Sibiu, 550024 Sibiu, Romania

**Keywords:** temporomandibular disorders, DC/TMD, Axis II, oral behaviors, Oral Behaviors Checklist, anxiety, depression, psychosocial factors, jaw function

## Abstract

*Background and Objectives*: Temporomandibular disorders (TMDs) are heterogeneous conditions whose clinical expression cannot be fully explained by local or structural findings alone. DC/TMD Axis II provides a psychobehavioral framework for assessing pain, disability, jaw functional limitation, psychological symptoms, and oral behaviors. This conceptual narrative review aimed to clarify how oral behaviors, anxiety, and depressive symptoms can be clinically interpreted together within the DC/TMD Axis II framework. *Materials and Methods*: A structured search was conducted in PubMed/MEDLINE and Scopus, with records exported in March 2026. Eligible English-language publications were limited to January 2001–March 2026. Google Scholar was used as a supplementary verification source. After deduplication, 2756 records were screened, 87 full-text reports were assessed, and 36 publications were included in the final narrative synthesis. Evidence was synthesized thematically and appraised according to study design, population, diagnostic framework, Axis II instruments, self-report reliance, confounding, and inferential strength. *Results*: Current literature supports associations between oral behaviors, anxiety, depressive symptoms, pain intensity, and mandibular functional limitation in TMD, especially in painful and functionally impaired profiles. These associations are not uniform across all TMD subtypes and are influenced by factors such as sex, pain burden, comorbidities, and psychosocial context. The Oral Behaviors Checklist is useful for standardizing self-reported oral behaviors, but its interpretation is limited by recall, awareness, and reporting bias. *Conclusions*: The reviewed evidence supports an interactional interpretation of oral behaviors, psychological symptoms, pain, and jaw function within the DC/TMD Axis II framework. However, because most available studies are cross-sectional and self-reported, this model should be understood as a clinically informed hypothesis supported by convergent associations, not as a confirmed causal pathway. Longitudinal and intervention-based studies are needed to clarify directionality, prognosis, and treatment-response relevance.

## 1. Introduction

Temporomandibular disorders (TMDs) represent a heterogeneous group of conditions involving the masticatory muscles, temporomandibular joints, and associated structures. Their clinical expression varies considerably, ranging from pain and joint sounds to restricted mandibular mobility and functional impairment, and this variability cannot be adequately explained solely by local examination or observable structural changes [1,2,3]. The frequent discrepancy between physical findings and patient-reported symptom burden has contributed to a shift from a predominantly biomechanical model to a biopsychosocial model in the understanding of TMD [1,2,3,4,5].

This shift is reflected in the Diagnostic Criteria for Temporomandibular Disorders (DC/TMDs), which organize assessments into two complementary axes [1,2]. Axis I addresses the physical and diagnostic dimension, while Axis II includes self-reported pain, disability, jaw functional limitation, psychological symptoms, and clinically relevant oral behaviors [1,2,6,7]. Therefore, Axis II is not a secondary component, but a necessary one for a more accurate description of the clinical and functional burden of the condition [1,2,6].

Among Axis II variables, anxiety and depressive symptoms, as well as waking-state oral behaviors, have received increasing attention in recent literature. Available studies suggest that these variables are associated with pain, functional limitation, disability, and reduced quality of life in TMD, particularly in painful forms [4,8,9,10,11,12,13]. At the same time, reported oral behaviors—such as awake clenching, repetitive tooth contact, or mandibular tension—are increasingly analyzed not merely as isolated habits, but as part of a broader psychobehavioral profile [14,15,16,17,18,19,20].

Although DC/TMD Axis II already provides an integrated psychobehavioral framework, a practical interpretive challenge remains. In clinical and research contexts, Axis II instruments are often reported as separate scores, and their combined meaning may be difficult to translate into clinical reasoning. The issue is therefore not the absence of conceptual integration within DC/TMD, but the need to clarify how oral behaviors, anxiety, depression, pain-related disability, and jaw functional limitation can be interpreted together without reducing them to a simple causal sequence. This is particularly important because much of the available evidence is cross-sectional and based on self-reported measures, which limits causal interpretation and makes it difficult to determine the temporal direction of these relationships [4,5,11,12,13,21,22].

The guiding question of this conceptual narrative review was: how can oral behaviors, anxiety, and depressive symptoms be clinically interpreted together within the DC/TMD Axis II framework, particularly in relation to pain-related disability and jaw functional limitation, without inferring unsupported causal pathways? Accordingly, the aim of this review was to provide a conceptual and clinically oriented synthesis of selected Axis II-related domains in TMD, focusing on oral behaviors, anxiety, and depression, while considering pain-related disability and jaw functional limitation as contextual dimensions of clinical burden. The review does not aim to validate the DC/TMD framework or to assess the psychometric reliability of its instruments; rather, it seeks to clarify how these variables can be interpreted together in clinical reasoning.

## 2. Methodological Considerations

### 2.1. Review Design

This article was designed as a conceptual narrative review with a clinical perspective. The aim was not to perform an exhaustive systematic review or meta-analysis, but to synthesize heterogeneous evidence relevant to the interpretation of oral behaviors, anxiety, and depression within the DC/TMD Axis II framework.

A conceptual narrative approach was considered appropriate because the topic involves interrelated diagnostic, psychometric, behavioral, psychological, functional, and clinical dimensions. Therefore, the objective was to integrate clinically relevant evidence and identify interpretive patterns rather than to provide a pooled quantitative estimate of effect.

The review was intended to support a comprehensive clinical interpretation of selected Axis II variables, with particular emphasis on oral behaviors, anxiety, and depression. Pain-related disability and jaw functional limitation were considered contextual Axis II dimensions relevant to the interpretation of the clinical burden associated with TMD.

### 2.2. Data Sources and Search Strategy

A structured literature search was conducted in PubMed/MEDLINE and Scopus, and the retrieved records were exported in March 2026. Google Scholar was used as a supplementary search source to verify potentially missed conceptual, psychometric, or citation-linked records. Eligible publications were limited to English-language articles published between January 2001 and March 2026. Because Scopus allows year-based filtering, the Scopus search was limited to publications from 2001 to 2026, and the export date in March 2026 was used as the upper temporal boundary for available records. This 25-year interval was selected to capture the transition from the RDC/TMD framework to the DC/TMD framework, as well as the subsequent development of Axis II-related instruments and biopsychosocial interpretations of TMD.

The search strategy was organized into five thematic blocks:

1. DC/TMD, RDC/TMD, and Axis II;
2. oral behaviors, awake oral behaviors, and the Oral Behaviors Checklist;
3. anxiety, depression, and psychological distress in TMD;
4. associations between oral behaviors and psychological symptoms;
5. pain-related disability and jaw functional limitation.

The PubMed/MEDLINE search used combinations of the following terms: “temporomandibular disorders”, “TMD”, “Diagnostic Criteria for Temporomandibular Disorders”, “DC/TMD”, “RDC/TMD”, “Axis II”, “oral behavior”, “oral behaviors”, “oral behaviour”, “oral behaviours”, “Oral Behaviors Checklist”, “Oral Behaviours Checklist”, “OBC”, “OBC-21”, “awake bruxism”, “parafunction”, “anxiety”, “GAD-7”, “depression”, “PHQ-9”, “psychological distress”, “jaw functional limitation”, “JFLS”, “pain-related disability”, and “Graded Chronic Pain Scale”.

The complete database-specific search strings, Boolean combinations, limits, search dates, and numbers of records retrieved or screened are reported in Supplementary Table S1.

### 2.3. Eligibility Criteria

Publications were considered eligible if they addressed at least one of the following dimensions: DC/TMD or RDC/TMD Axis II assessment; oral behaviors or the Oral Behaviors Checklist; anxiety, depression, or psychological distress in TMD; pain-related disability; jaw functional limitation; or the conceptual interpretation of TMD within a biopsychosocial or psychobehavioral framework.

Eligible sources included foundational DC/TMD and RDC/TMD papers, observational studies, psychometric validation studies, systematic reviews, meta-analyses, narrative or conceptual reviews, and clinically relevant consensus or framework papers. Studies were included only if they were published in English, fell within the defined time interval, and were directly relevant to the objective of the review.

Publications were excluded if they focused exclusively on imaging, biomarkers, occlusion, orthodontic parameters, or treatment efficacy without relevant Axis II content. Articles were also excluded if they were not published in English, lacked direct relevance to the review objective, were duplicates, or did not provide sufficient methodological or conceptual information for interpretation.

### 2.4. Study Selection and Source Prioritization

Titles and abstracts were screened independently by two authors. Potentially relevant publications were assessed in full text. Disagreements regarding inclusion were resolved through discussion until consensus was reached.

The five PubMed/MEDLINE search blocks retrieved 2432 records before deduplication. After PMID-based removal of 688 duplicate records across PubMed/MEDLINE search blocks, 1744 unique PubMed/MEDLINE records remained.

The five Scopus search blocks retrieved 3463 records before deduplication. After EID-, DOI-, and normalized title-year-based removal of 1003 duplicate records across Scopus search blocks, 2460 unique Scopus records remained.

Cross-database deduplication was then performed using DOI matching, followed by normalized title-and-year matching for records without a shared DOI. This process identified 1448 Scopus records that duplicated PubMed/MEDLINE records. Therefore, 2756 unique PubMed/MEDLINE and Scopus records were screened by title and abstract.

Google Scholar was used as a supplementary search source to verify whether relevant conceptual, psychometric, or citation-linked sources had been missed by the PubMed/MEDLINE and Scopus searches. Six predefined Google Scholar queries were used, and the first 100 relevance-ranked results for each query were screened, as reported in Supplementary Table S1. Because Google Scholar output is ranking-dependent and less reproducible than database searches, these records were not merged quantitatively with the PubMed/MEDLINE and Scopus dataset. No additional unique eligible source was identified through Google Scholar beyond the records already retrieved from PubMed/MEDLINE and Scopus.

After title and abstract screening, 2669 records were excluded because they did not meet the eligibility criteria or were not directly relevant to the conceptual and clinical objectives of the review. Eighty-seven publications were retained and assessed in full text.

Of these, 51 full-text reports were excluded for one or more of the following reasons: limited relevance to DC/TMD Axis II; lack of direct analysis of oral behaviors, anxiety, depression, pain-related disability, or jaw function; treatment-focused content without relevant Axis II interpretation; predominantly imaging, occlusion, biomarker, or biomechanical focus without psychosocial or behavioral relevance; conceptual overlap with more comprehensive sources already included in the synthesis; or insufficient methodological or clinical relevance for the final synthesis.

Thirty-six publications were included in the final narrative synthesis. Of these, 15 key empirical, psychometric, and review studies are summarized in Table 1. During revision, five additional contextual sources were incorporated to address reviewer-requested biopsychosocial modifiers, namely sex differences and pandemic-related context. These sources were used to contextualize the clinical interpretation of Axis II variables and were not included in the core selection flow or in Table 1.

**Figure 1:**
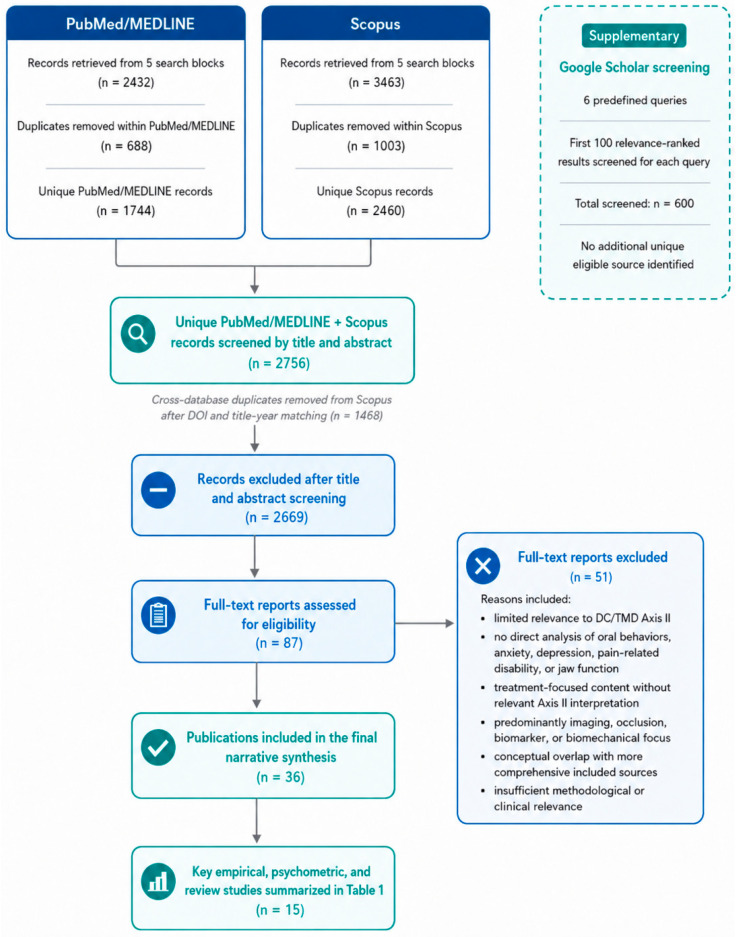
Literature identification and selection flow diagram.

The literature identification and selection process is summarized in Figure 1.

### 2.5. Evidence Weighting and Appraisal

Because this was a conceptual narrative review, no formal risk-of-bias tool was applied. However, the included literature was appraised using predefined interpretive criteria: study design, population characteristics, diagnostic framework used for TMD, use of validated Axis II instruments, reliance on self-reported outcomes, control of confounding factors, clinical relevance, and ability to support causal, prognostic, or associative interpretations.

Different types of evidence were not treated as equivalent. Foundational DC/TMD and RDC/TMD papers were used to define the diagnostic and assessment framework. Psychometric studies were used to evaluate the validity, reliability, and limitations of instruments such as the OBC, GAD-7, PHQ-9, JFLS, and GCPS. Systematic reviews and meta-analyses were considered more informative for broad associations. Cross-sectional studies were interpreted as evidence of association rather than causality. Conceptual papers were used to support clinical framing and interpretation, but not as primary evidence for empirical associations.

This distinction was essential because the included sources contributed to different levels of interpretation. Foundational and conceptual papers supported the diagnostic and biopsychosocial framework; psychometric studies supported the appropriateness and limitations of Axis II-related instruments; systematic reviews and meta-analyses informed broader patterns of association; and observational studies provided clinical evidence of relationships between oral behaviors, psychological symptoms, pain, and jaw function. Therefore, no single category of evidence was used alone to substantiate the proposed interpretive model.

Particular caution was applied when interpreting evidence based on self-report, cross-sectional designs, heterogeneous TMD populations, or studies without adequate control for confounding variables such as chronic pain, sleep disturbances, migraine, widespread pain, or central sensitization.

### 2.6. Synthesis Approach

Evidence was synthesized thematically, with interpretation based on convergence across evidence types rather than on the findings of any single study design. The evidence was synthesized thematically. The synthesis was organized around three central domains: oral behaviors, anxiety, and depression. Pain-related disability and jaw functional limitation were interpreted as clinically relevant contextual Axis II variables that help clarify the functional and symptomatic burden of TMD.

The synthesis focused on identifying convergent patterns across the literature rather than establishing causal pathways. Therefore, the proposed interactional model linking oral behaviors, emotional vulnerability, pain, and mandibular function was interpreted as a clinically informed hypothesis supported by convergent associations, not as a confirmed causal mechanism.

This approach was intended to support an integrated clinical interpretation of Axis II variables while avoiding two opposite errors: reducing TMD to a purely biomechanical condition or over-psychologizing the clinical case without sufficient integration of physical, functional, and psychosocial information.

## 3. Conceptual Framework: DC/TMD and the Significance of Axis II

The DC/TMD currently represents the reference framework for the standardized assessment of temporomandibular disorders, both in research and in clinical practice [1,2]. One of its main strengths is that it operationalizes the biopsychosocial model, avoiding the reduction of TMD to a purely local pathology or to an exclusively structural interpretation [1,2,5]. Thus, clinical evaluation is not limited to the identification of a physical diagnosis, but also includes the way pain is experienced, how function is affected, and how symptomatology is influenced by the patient’s psychobehavioral context [1,2,6,7].

Axis I is oriented toward the physical and diagnostic dimension, encompassing the main TMD subtypes based on patient history and standardized clinical examination [1,2]. However, Axis I alone cannot explain why patients with comparable somatic presentations report different levels of pain, disability, or functional impact [1,2,6]. This discrepancy helps explain why the Axis II assessment is clinically relevant.

The emphasis on Axis II should not be understood as a dismissal of physical, occlusal, or biomechanical considerations in TMD assessment. Rather, it reflects the need to avoid reducing TMD symptoms to occlusal findings alone. The role of occlusion in TMD remains clinically debated, with contemporary literature suggesting that occlusal factors should be interpreted cautiously and within a broader diagnostic and functional context [23,24]. Similarly, concepts such as centric relation have been critically revisited, with emphasis on their limited explanatory value when used in isolation for complex orofacial pain presentations [25]. From this perspective, Axis II complements rather than replaces the physical and functional assessment of TMD.

Axis II includes the assessment of self-reported pain, jaw functional limitation, psychological distress, anxiety and depressive symptoms, and clinically relevant oral behaviors [1,2,6,7]. Comparisons between the instruments used in RDC/TMD and those later incorporated into DC/TMD have supported the consistency and methodological validity of this axis, reinforcing the idea that the description of a patient with TMD cannot be complete in the absence of a psychosocial and behavioral assessment [6].

The clinical importance of Axis II does not lie in the “psychologization” of TMD, but in its ability to provide a more realistic description of the clinical burden. A patient with TMD is defined not only by the diagnostic category, but also by pain intensity, degree of functional impairment, level of distress, and associated behavioral patterns [4,5,6,7,21]. From this perspective, Axis II provides the logical framework within which the relationship between oral behaviors, anxiety, and depression can be analyzed in an integrated manner.

However, for the purposes of the present review, DC/TMD Axis II should not be interpreted as a self-sufficient explanatory model. Axis II instruments quantify patient-reported pain, functional limitation, psychological symptoms, and behavioral patterns, but they do not establish causal mechanisms and do not replace Axis I diagnosis or clinical judgment. Their value depends on their integration with physical findings, pain history, functional status, comorbidities, and the broader clinical context.

Therefore, this review uses DC/TMD Axis II primarily as an organizing framework for selected psychobehavioral domains, rather than as evidence that oral behaviors, anxiety, or depression independently explain TMD. This distinction is important because elevated Axis II scores may indicate clinical burden and complexity, but they should not be interpreted as proof of etiology, prognosis, or treatment response without longitudinal or intervention-based evidence.

## 4. The Psychological Dimension in TMD: Anxiety and Depression

Anxiety and depressive symptoms occupy a central place in the Axis II literature, as they are frequently associated with TMD, particularly with painful forms and those involving greater functional impairment [4,9,10,11,12,13,21]. Recent systematic reviews and meta-analyses converge in showing that anxiety, depression, and other psychological factors are significantly associated with both the presence and severity of TMD, although the strength of these relationships varies depending on study design, the population investigated, and the diagnostic subtype analyzed [4,9,12].

Importantly, the association between anxiety, depression, and TMD does not appear to be uniform across all diagnostic subtypes. A systematic review and meta-analysis by Reis et al. reported that anxiety and depression were more frequent and more severe among patients with myofascial pain than among patients with other TMD subtypes. This finding is relevant to the present review because it supports a subtype-sensitive interpretation of Axis II variables. Psychological symptoms should therefore be interpreted in relation to the clinical TMD phenotype, particularly the presence of muscular pain, rather than generalized to all TMD presentations in the same way [26].

Anxiety appears to be one of the most consistent variables associated with TMD. Studies examining psychosocial profiles in TMD have shown that patients with higher anxiety scores tend to report more intense pain, greater functional limitation, and higher levels of distress [10,11,13,21]. Moreover, general anxiety and health-related anxiety seem to correlate not only with pain, but also with sleep, jaw function, and oral behaviors, suggesting the presence of a broader clinical profile rather than an isolated relationship [11,13,21].

Depression is likewise frequently associated with TMD, particularly in painful subgroups and in chronic conditions [10,12,13]. A recent meta-analysis suggested an association between depression and the risk of developing TMD; however, the interpretation of this relationship remains cautious, as depression may reflect both pre-existing emotional vulnerability and a consequence of persistent pain and functional disability [12]. Other studies have reported correlations between depressive symptoms and pain intensity, impaired jaw function, sleepiness, and reduced oral health-related quality of life [10,13].

The mechanisms through which anxiety and depression are associated with TMD are likely multifactorial. The literature points to altered descending inhibitory control, central sensitization, shared neural networks involved in pain processing and affect regulation, as well as bidirectional relationships between pain and mental health [5,21,22]. Within this framework, psychological symptoms should be considered neither as sole explanations nor as simple secondary reactions, but as components of a biopsychosocial profile in which pain, function, and emotional vulnerability interact dynamically.

Importantly, the current literature does not support a simple causal relationship between anxiety, depression, and TMD. The predominance of cross-sectional studies and methodological heterogeneity limits direct comparability of results and calls for caution in interpretation [4,9,12,21]. What can be reasonably supported is the clinical relevance of these variables within the TMD patient profile, rather than a unidirectional direction of effects.

## 5. Oral Behaviors as a Behavioral Dimension of Axis II

Oral behaviors represent a broad group of repetitive functional manifestations involving the mandible, orofacial musculature, dental contact, or the use of oral structures beyond strictly necessary functions [14,15,16,17,18,19,20,27]. These include, among others, awake tooth clenching, repetitive tooth contact, mandibular tension, or maintaining the mandible in an unusual position [14,15,16,17,18,19,20]. Current literature emphasizes that the mere presence of such behaviors does not automatically equate to pathology. Clinical relevance arises especially when their frequency, persistence, or context transforms them into potential contributors to overload, maintenance of symptoms, or markers of an unfavorable functional profile [16,17,18,19,20,27].

Interest in oral behaviors stems from their potential to increase the functional load on the stomatognathic system. When repeated outside an efficient functional pattern, they may contribute to muscular overload, unnecessary tooth contact, and inefficient mandibular use [14,16,17,18,19,20]. Several studies have shown that higher oral behavior scores are associated with painful TMD, chronic pain, impaired jaw function, and psychological distress [14,16,17,18,19,20,28]. However, this association should not be interpreted simplistically. Oral behaviors may function both as maintaining factors of symptoms and as behavioral expressions of pain, hypervigilance, or distress [16,17,18,19,20,28].

Within the DC/TMD framework, oral behaviors logically belong to Axis II, as they describe a behavioral dimension that cannot be directly inferred from the physical examination [1,2,6]. The most used instrument for their assessment is the Oral Behaviors Checklist (OBC), a 21-item self-report instrument included in the DC/TMD Axis II assessment and introduced to standardize the self-reporting of clinically relevant mandibular and dental behaviors [1,14,15]. In this review, OBC is used as the general abbreviation for the Oral Behaviors Checklist, while OBC-21 refers specifically to its 21-item format; the two terms do not indicate different instruments. Early studies supported the reliability of the OBC in assessing targeted waking-state behaviors, while subsequent research demonstrated correlations between OBC scores and facial pain, ecological momentary assessment, and other relevant clinical indicators [14,15,29,30].

The utility of the OBC lies in its ability to transform an otherwise imprecisely described dimension of everyday behavior into information that is comparable across patients and studies [14,15,29]. In addition, Vlad et al. [16] used the OBC-21 to describe patterns of self-reported oral behaviors in a Romanian adult population, supporting the contextual utility of standardized behavioral screening outside strictly clinical TMD samples; however, because that study did not directly assess TMD patients or psychological variables, it is considered here only as contextual evidence. However, the OBC does not provide an autonomous diagnosis and cannot determine on its own whether a behavior is pathological. As a self-report instrument, its results depend on the patient’s level of awareness, recall capacity, and interpretation of the behaviors being assessed [30,31]. Moreover, patients’ beliefs that certain habits are harmful may influence their reporting, introducing potential cognitive bias [31].

The distinction between oral behaviors and bruxism is also essential. Recent literature supports the view that bruxism should be understood as a behavior rather than a disorder per se, and instruments such as the Standardized Tool for the Assessment of Bruxism (STAB) aim to provide a more specific evaluation [27,32]. Nevertheless, the OBC remains valuable for Axis II analysis, as it captures a broader spectrum of oral and mandibular behaviors than bruxism alone [14,27,32].

## 6. The Relationship Between Oral Behaviors, Anxiety, and Depression

Available literature suggests that behavioral and psychological dimensions do not function independently in Temporomandibular Disorders (TMDs); rather, they tend to cluster within the same clinical profile, characterized by higher pain intensity, more pronounced functional limitation, and increased distress [1,4,6,21]. The biopsychosocial model and the logic of Axis II support an integrated interpretation, in which the patient’s demand on the stomatognathic system, their perception of pain, and their emotional vulnerability are part of a common network of interactions [1,4,5,21].

### 6.1. Oral Behaviors and Anxiety

Available data support an association between self-reported oral behaviors and anxiety symptoms. A cross-sectional study conducted on TMD patients in China demonstrated that OBC scores correlate significantly with GAD-7 scores and mandibular functional limitation [11]. Other studies have reported convergent results, highlighting associations between waking oral behaviors, generalized anxiety, health anxiety, and psychological distress [13,19,21,28].

The plausibility of this relationship is supported by the fact that anxiety can promote somatic hypervigilance, increased muscle tension, symptom focusing, and repetitive mandibular use patterns [5,21,22]. Concurrently, the presence of pain and dysfunction can amplify health-related anxiety and excessive monitoring of the orofacial region, making it difficult to distinguish a primary cause from a secondary effect [13,21,22]. Consequently, the available evidence is more consistent with an interactional interpretation than with a simple linear explanation, although causal direction cannot be established from the current literature.

### 6.2. Oral Behaviors and Depression

The relationship between oral behaviors and depression appears less direct than the relationship with anxiety and seems more dependent on the context of persistent pain and functional impairment. In studies simultaneously analyzing OBC, PHQ-9, and other Axis II variables, depressive symptoms frequently appeared in association with profiles characterized by more intense pain, poorer mandibular function, and a higher frequency of oral behaviors [10,11,13,19]. Compared to anxiety, the association between depression and oral behaviors seems more strongly influenced by the overall clinical severity of the case and the cumulative symptomatic burden [10,12,13,21].

This observation is consistent with data showing that depression holds particular relevance in painful TMD and chronic contexts [4,12]. Oral behaviors may contribute to the maintenance of discomfort and pain, while persistent pain can promote mood disturbances and functional withdrawal. From this perspective, depression should be interpreted neither as a sole determinant of oral behaviors nor merely as a late-stage effect, but rather as part of the same clinical cycle in which pain, function, and distress can mutually amplify one another [4,5,12,21].

### 6.3. Pain and Functional Limitation as Contextual Axis II Dimensions

Pain intensity and mandibular functional limitation are central to the interpretation of the relationship between oral behaviors and psychological symptoms. Several studies have shown that patients with higher pain intensity and greater functional limitation also tend to present elevated scores for anxiety, depression, and oral behaviors [10,11,13,19,21].

This interpretation is clinically relevant because elevated OBC, GAD-7, or PHQ-9 scores should not be read as isolated findings. Their meaning depends on the patient’s pain burden, mandibular function, comorbid conditions, and broader clinical context. The literature on central sensitization further indicates that associations between Axis II variables may be influenced by migraine, sleep disturbances, widespread pain, or parafunctions, underlining the need to interpret these variables as part of a broader clinical profile rather than as independent causal factors [21,22,28].

### 6.4. Synthesis: An Interactional Interpretative Model

Taken together, the evidence reviewed in the preceding subsections supports an interactional interpretation rather than a unidirectional explanatory model. Oral behaviors, anxiety, depressive symptoms, pain intensity, and mandibular functional limitation appear to cluster within clinically more complex TMD profiles, especially in painful cases and in patients with greater functional impairment [10,11,13,19,21,28]. However, the available literature does not establish whether psychological symptoms precede oral behaviors, whether oral behaviors contribute to symptom persistence, or whether both reflect the broader burden of pain and dysfunction.

Therefore, the proposed interactional model should be understood as a clinically informed hypothesis supported by convergent associations, not as a confirmed causal pathway. Its main value is interpretative: it helps explain why Axis II variables should be evaluated together and in relation to pain and jaw function, rather than treated as separate or independent findings. This model does not imply that oral behaviors, anxiety, or depression independently cause TMD, nor that elevated Axis II scores can by themselves predict prognosis or treatment response.

Table 1 is illustrative rather than exhaustive and summarizes 15 key empirical, psychometric, and review studies selected from the 35 publications included in the final narrative synthesis. These studies were selected because of their direct relevance to oral behaviors, anxiety, depression, pain-related disability, jaw functional limitation, or Axis II-related assessment in TMD; their use of validated or clinically relevant instruments; and their contribution to the interpretation of psychobehavioral associations within the DC/TMD Axis II framework.

**Table 1:** Illustrative summary of key studies on oral behaviors, psychological symptoms, and Axis II-related variables in TMD.

Study	Design and Sample Size	Participant Characteristics	Instruments/Measures	Key Findings/Effect Measures	Main Limitations
Yap et al., 2025 [10]	Observational study; *n* = 798 TMD patients	Chinese adult TMD patients; mean age 29.8 ± 10.7 years; 79.6% women	PHQ-9, GAD-7, DC/TMD measures, oral behavior, pain, sleep, jaw function, OHRQoL tools	Comorbid depression/anxiety was associated with higher pain, sleep propensity, oral behaviors, jaw dysfunction, and poorer OHRQoL. In adjusted models, depression alone was associated with combined TMD (OR = 2.26; 95% CI: 1.19–4.27), and comorbid depression/anxiety with pain-related TMD (OR = 1.72; 95% CI: 1.02–2.89) and combined TMD (OR = 1.78; 95% CI: 1.14–2.77).	Cross-sectional design; single-site Chinese sample; predominance of women; self-reported psychosocial and behavioral measures
Xu et al., 2021 [11]	Cross-sectional study; *n* = 537 TMD patients	Chinese TMD patients; mean age 31.55 ± 12.08 years; 84.0% women	OBC, JFLS, GAD-7, PHQ-9	OBC scores correlated with PHQ-9 (r = 0.375, *p* < 0.01) and GAD-7 (r = 0.322, *p* < 0.01); OBC also correlated significantly with JFLS. OBC internal consistency: Cronbach’s α = 0.771.	Cross-sectional design; self-reporting; convenience clinical sample; no healthy control group
Hoff et al., 2025 [12]	Systematic review with meta-analysis; secondary evidence	Studies comparing depression across RDC/TMD or TMD diagnostic groups	Depression measures; RDC/TMD/TMD diagnostic groups	Depression was a significant risk factor for RDC/TMD Axis I muscle disorders and arthralgia/osteoarthritis/osteoarthrosis, but not for disc displacement. Severe depression was associated with approximately fourfold higher risk of TMD compared with moderate depression.	Heterogeneity among primary studies; differences in diagnostic criteria and depression measures; causal inference remains limited
Yap et al., 2025 [13]	Observational study; *n* = 371 TMD patients	TMD patients; mean age 29.8 years; 79.5% women	GAD-7, Whiteley Index-8, PHQ-9, DC/TMD Axis II measures, pain, jaw function, OHRQoL	Moderate-to-severe general anxiety and depression were each present in 15.1%; high health anxiety in 19.7%. General anxiety was strongly correlated with health anxiety and depression; health anxiety was moderately correlated with depression and OHRQoL (rs = 0.60–0.77). Low OHRQoL odds increased with combined TMD (OR = 2.86), general anxiety (OR = 1.35), health anxiety (OR = 1.13), pain intensity (OR = 1.03), and jaw functional limitation (OR = 1.04).	Cross-sectional design; self-report measures; single clinical population; limited causal interpretation
Zhong et al., 2024 [17]	Retrospective case–control study; *n* = 280 adult women	210 women with TMD and 70 non-TMD controls	OBC-Ch, GAD-7, PHQ-9, TMD subgroup classification	OBC scores differed across TMD subgroups and were higher in TMD groups than controls. Sleep-related OBC scores were higher in all TMD subgroups than controls (*p* < 0.001). Pain-related and combined TMD groups also showed higher GAD-7 and PHQ-9 scores, supporting subgroup-specific psychobehavioral profiles.	Retrospective design; women-only sample; limited generalizability; associative findings
Donnarumma et al., 2021 [18]	Observational study; *n* = 743	Non-TMD and TMD subgroups: dysfunctional TMD, painful TMD, and painful-dysfunctional TMD	OBC; DC/TMD subgroup classification	OBC analysis identified two behavioral dimensions: non-functional and functional activities. OBC total and subscale scores differed across TMD subgroups, with non-functional activities more prominent in painful and painful-dysfunctional TMD profiles.	Cross-sectional/observational design; associations do not establish causality; subgroup differences may depend on scoring method
Yu et al., 2025 [19]	Cross-sectional observational study; *n* = 207	96 TMD patients and 111 non-TMD controls	OBC, JFLS, PHQ-9, GAD-7, PHQ-15, chronic pain measures	TMD patients had higher OBC, PHQ-9, GAD-7, and PHQ-15 scores than controls. OBC: 20.96 ± 6.10 vs. 14.32 ± 7.95, *p* < 0.001; PHQ-9: 9.93 ± 4.03 vs. 4.77 ± 4.42, *p* < 0.001; GAD-7: 8.16 ± 3.27 vs. 2.97 ± 3.38, *p* < 0.001. In TMD patients, OBC correlated with depression and anxiety (r = 0.52 for both), and with chronic pain (rs = 0.23–0.28). Chronic pain showed significant indirect effects in the relationship between oral behaviors and psychological outcomes.	Cross-sectional design; moderate sample size; self-reported measures; mediation findings require longitudinal confirmation
Hoa et al., 2025 [20]	Cross-sectional study; *n* = 120 TMD patients	Patients with temporomandibular joint disorders/TMD-related pain profiles	OBC, VAS pain intensity, PHQ-9, GAD-7, GCPS, PHQ-15, JFLS-8	After multivariable adjustment, pain intensity remained independently associated with total OBC scores (coefficient = 1.829; 95% CI: 0.51–3.15; *p* = 0.007). Anxiety was independently associated only with nocturnal bruxism (OR = 2.95; 95% CI: 1.30–6.67; *p* = 0.010), while depression showed no independent association.	Cross-sectional design; modest sample size; self-reporting; limited generalizability
Assiri, 2024 [21]	Cross-sectional analytical study; *n* = 120 pain-related TMD patients	60 women and 60 men diagnosed with pain-related TMD according to DC/TMD Axis I	NEO-FFI, GCPS v2.0, PHQ-4, GAD-7	High-intensity characteristic pain was reported by 49.2%; pain-related disability >30 days and disability score ≥70 by 14.2%; severe distress by 16.7%; severe anxiety by 18.3%. Female sex and personality factors were associated with higher DC/TMD Axis II psychosocial impairment scores (*p* < 0.05).	Cross-sectional design; single pain-related TMD subtype; personality assessment not part of standard DC/TMD Axis II; limited generalizability
Calixtre et al., 2024 [22]	Cross-sectional telehealth study; *n* = 95 women	42 women with painful TMD and 53 without TMD	CSI, TMD assessment, migraine, depression, widespread pain, parafunctional oral habits	The association between CSI scores and painful TMD was influenced by migraine, depressive symptoms, widespread pain, and parafunctional oral habits, indicating that central sensitization-related findings may be confounded by comorbid pain and psychosocial variables.	Cross-sectional design; telehealth assessment; women-only sample; confounding limits direct interpretation
van der Meulen et al., 2014 [14]	Validation study; *n* = 155 TMD patients	Dutch TMD patients; 77% women; mean age 43.6 ± 14.4 years	Dutch OBC, Oral Parafunctions Questionnaire, facial pain measures	Dutch OBC showed good test–retest reliability (ICC = 0.86, *p* < 0.001) and concurrent validity with the Oral Parafunctions Questionnaire (r = 0.757, *p* < 0.001). No significant correlation was found between OBC scores and facial pain intensity (r = 0.069, *p* = 0.892).	Validation study; self-reporting; limited support for direct pain association
Markiewicz et al., 2006 [15]	Reliability study; *n* = 54	27 TMD cases and 27 controls	OBC behavioral terms; surface EMG of masseter, temporalis, suprahyoid, and biceps muscles	EMG-based test–retest reliability for targeted waking-state oral behaviors generally ranged from 0.60 to 0.98 across muscle groups. Elevator muscle reliability was higher in TMD cases than controls.	Laboratory-based behavioral performance; small sample; not a clinical outcome study
Kaplan and Ohrbach, 2016 [29]	Ecological assessment and reliability study; *n* = 22 for EMA component; *n* = 74 for OBC test–retest component	Participants assessed for waking-state oral parafunctional behaviors in natural environment and by questionnaire	OBC, ecological momentary assessment (EMA)	OBC pre/post reliability was moderate to good; self-reported OBC behavior frequencies showed variable correspondence with EMA. Reported EMA/OBC associations varied by behavior, supporting OBC utility but also highlighting limits of retrospective self-report.	Small EMA sample; ecological assessment is more difficult to implement clinically; self-report/EMA agreement varies by behavior
van Selms et al., 2020 [31]	Observational study; n = 329	DC/TMD myalgia patients; 82.4% women; mean age 41.9 ± 14.7 years	Self-reported waking-state oral behaviors, orofacial pain, belief about harmfulness, somatic symptoms, depression, anxiety	The association between self-reported waking-state oral behaviors and orofacial pain depended on patients’ belief that these behaviors were harmful. This supports the role of cognitive appraisal/reporting bias in OBC–pain associations.	Observational design; self-reporting; cognitive belief effects may influence symptom reporting
Emodi-Perlman et al., 2025 [30]	Non-patient student study; *n* = 118	34 participants with TMD-related pain symptoms and 84 without TMD-related pain symptoms	OBC and EMA	Participants with TMD-related pain symptoms performed more waking-state oral behaviors. EMA model: teeth grinding increased odds of TMD-related pain symptoms by 22%; Nagelkerke R^2^ = 0.232. OBC model: teeth grinding increased odds by 85%, holding/jutting the jaw by 82%, and clenching by 67%; Nagelkerke R^2^ = 0.468.	Non-clinical population; cross-sectional design; self-report and EMA may capture different behavioral dimensions

## 7. Clinical Interpretation and Practical Implications of Axis II

One of the primary utilities of Axis II is that it assists the clinician in more accurately interpreting the frequent discrepancy between the physical examination and the patient’s self-reported suffering. Patients with comparable somatic findings may exhibit vastly different levels of pain, disability, and need for intervention; these differences become more intelligible when psycho-behavioral variables are integrated [1,2,6,7,21].

In this context, the OBC, GAD-7, and PHQ-9 are valuable as screening and quantification tools but must not be interpreted in isolation. An elevated OBC score does not, in itself, demonstrate a causal mechanism, nor does it establish whether a behavior is pathological. Similarly, high scores on the GAD-7 or PHQ-9 do not automatically categorize a case as predominantly “psychological,” nor do they invalidate the relevance of the physical examination. Proper clinical interpretation is an integrated one, in which pain, mandibular function, emotional symptoms, and oral behaviors are evaluated as components of the same Axis II profile.

The principal instruments relevant to Axis II and their clinical utility in the context of TMD are summarized in Table 2.

This perspective supports the necessity for more integrated clinical frameworks for interpreting questionnaire data, avoiding a fragmented reading of the results. The practical value lies not in the simple identification of elevated scores, but in their use for describing clinical burden, guiding a more comprehensive assessment, and identifying patients in whom psycho-behavioral factors may be relevant to symptom interpretation. Their prognostic value, including relevance to symptom persistence, adherence, or treatment response, should be interpreted cautiously and requires further longitudinal and intervention-based evidence.

### Sex and Pandemic-Related Context as Biopsychosocial Modifiers

Sex should be considered when interpreting the relationship between oral behaviors, psychological symptoms, pain, and jaw function in TMD. Epidemiological evidence indicates female predominance in TMD, with a systematic review and meta-analysis reporting that women have approximately twice the risk of developing TMD compared with men [36]. This aspect is relevant to the present synthesis because many studies on TMD, anxiety, depression, and oral behaviors include predominantly female samples, which may influence both symptom prevalence and reporting patterns. Recent mechanistic literature also suggests that sex differences in TMD may involve multiple biological and psychosocial mechanisms, including hormonal regulation, craniofacial anatomy, pain modulation systems, and comorbidity with migraine [37]. Therefore, sex should not be treated only as a demographic descriptor, but also as a potential contextual or moderating factor in Axis II interpretation.

The COVID-19 pandemic represents another relevant psychosocial context for interpreting recent literature on TMD, bruxism, oral behaviors, anxiety, and depression. Pandemic-related stress, uncertainty, social isolation, and health-related anxiety have been associated with increased psychological distress and may have influenced the reporting or aggravation of TMD and bruxism-related symptoms [38,39]. A systematic review on COVID-19-related anxiety, stress, depression, TMD, and headaches reported associations between pandemic-related psychological distress and increased TMD symptoms, although the evidence base was limited and heterogeneous [38]. Similarly, cross-national research conducted during lockdown periods suggested that anxiety, depression, personal concerns, and female sex were associated with the presence or worsening of TMD and bruxism symptoms in some populations [39]. These findings should be interpreted cautiously, as most pandemic-related studies are cross-sectional and context-dependent. However, they support the broader view that Axis II variables are sensitive to psychosocial stressors and should be interpreted within their temporal and social context.

In a Romanian dental emergency setting, Moca et al. reported changes in dental emergency service use during the COVID-19 lockdown, with an increase in emergency visits during March–May 2020 compared with the corresponding periods in 2019 and 2021 [40]. Although this study did not assess TMD, oral behaviors, or Axis II variables directly, it provides contextual evidence that the pandemic altered dental care-seeking patterns and the clinical environment in which dental and orofacial complaints were managed [40]. For this reason, it is considered here only as contextual evidence, not as direct support for the relationship between TMD, oral behaviors, anxiety, or depression.

Clinically, two interpretive risks should be avoided. The first is reducing TMD to an exclusively mechanical problem, with insufficient attention to pain, distress, and oral behaviors [1,4,5]. The second is overemphasizing psychological factors without adequate consideration of physical and functional findings [1,2,6]. In cases involving persistent pain, marked functional limitation, and a high-load psycho-behavioral profile, an interdisciplinary approach may be clinically justified; however, this decision must result from an integrated case evaluation rather than from scores interpreted in isolation [4,5,21,41].

## 8. Limitations of Available Literature

The literature regarding the relationship between oral behaviors, anxiety, and depression in TMD presents several significant methodological limitations. First, most available studies employ a cross-sectional design, which complicates the establishment of temporal relationships and precludes the formulation of robust causal conclusions [4,9,11,12,13,19,21]. While reported associations may be clinically relevant, the sequence in which these variables emerge and mutually influence each other remains largely unclear [4,5,20].

Second, many studies rely on self-reporting for oral behaviors as well as for psychological and functional symptoms [11,13,14,15,16,29,30,31]. This dependence on self-reporting introduces potential recall bias, variations in semantic interpretation, and the influence of patient beliefs on responses [30,31]. Although the OBC, GAD-7, and PHQ-9 are useful and validated instruments, they remain tools for screening and quantification, rather than complete substitutes for an integrated clinical evaluation [14,15,29,30,31,32,33,34,35].

Third, there is considerable diagnostic and methodological heterogeneity. TMD subtypes are not always rigorously separated, study populations vary by age, sex, severity, and clinical context, and the instruments used are not uniform across studies [10,11,12,13,19,21,22]. Additionally, variables such as migraines, sleep, widespread pain, or central sensitization may act as confounding factors and influence the observed relationships [21,22]. For these reasons, current literature supports the clinical relevance of Axis II but does not justify the transformation of repeated associations into simplistic explanations.

## 9. Limitations of the Present Review

The current article has the inherent limitations of a narrative review. The selection of literature was guided by conceptual and clinical relevance to the analyzed topic, rather than the formal exhaustiveness of a systematic review. Consequently, the final synthesis remains influenced by the authors’ thematic choices, even though these were made transparently and based on a structured search.

The restriction to English-language publications may have introduced language-selection bias and may have led to the exclusion of relevant studies published in other languages.

In addition, although Google Scholar was used only as a supplementary verification source, its ranking-dependent output and limited reproducibility remain methodological limitations. To reduce this limitation, predefined queries were used and the first 100 relevance-ranked results were screened for each query; however, no additional unique eligible source was identified through Google Scholar.

Furthermore, this review prioritized literature regarding waking oral behaviors and their evaluation via the OBC; this means that other dimensions of bruxism, alternative psychometric instruments, or other related fields were discussed only to the extent necessary to clarify the central argument. Additionally, specific treatments, biomarkers, neuroimaging, and broad debates regarding occlusion were not developed in detail, as they did not represent the core of the present analysis.

## 10. Conclusions

Current literature supports the clinical relevance of Axis II psycho-behavioral variables in TMD. Anxiety symptoms, depressive symptoms, and oral behaviors are frequently reported within clinical profiles characterized by increased pain and functional impairment. Oral behaviors evaluated through the OBC appear to be associated with anxiety and jaw functional limitation, while their relationship with depression seems more evident in the context of persistent pain and functional disability.

However, the available evidence does not support a simple, unidirectional causal relationship between these dimensions. Much of the current literature is cross-sectional and relies substantially on self-reported measures. Therefore, the proposed interactional model should be interpreted as a clinically informed hypothesis supported by convergent associations, rather than as a confirmed explanatory pathway. The most prudent interpretation is that pain, oral behaviors, mandibular function, and emotional vulnerability are interrelated within a complex biopsychosocial profile.

The main contribution of this perspective is that it supports the joint interpretation of OBC, GAD-7, and PHQ-9 scores, rather than their isolated use. The Axis II assessment may help describe clinical burden and guide a more comprehensive evaluation of patients with TMD, but its prognostic or treatment-response value requires further longitudinal and intervention-based evidence. Future studies using longitudinal designs, ecological assessments, and more homogeneous methodological approaches are needed to clarify temporal direction, clinical relevance, and therapeutic implications.

## Figures and Tables

**Table 2:** Relevant Axis II instruments and their clinical utility in TMD.

Instrument	Evaluated Domain	Clinical Utility	Main Limitation	Relevance for This Review
OBC (21-item format) [14,15,29,30]	Waking oral behaviors	Quantifies reported mandibular and dental behaviors in a comparable manner	Does not establish pathology; relies on awareness and recall	Central to the analysis of the behavioral component
GAD-7 [33]	Anxiety symptoms	Rapid and validated screening for anxiety	Does not replace a clinical psychological evaluation	Useful for the relationship between anxiety, pain, and oral behaviors
PHQ-9 [34,35]	Depressive symptoms	Brief and validated screening for depression	Does not independently explain the clinical severity of the case	Useful for discussing depression within the painful TMD profile
JFLS (Jaw Functional Limitation Scale) [8]	Mandibular functional limitation	Quantifies the functional impact of symptoms	Variability between studies and versions	Important for interpreting the link between symptoms and function
Pain-related disability and psychological distress measures [1,2,6]	Functional and psychosocial burden	Completes the Axis II clinical picture	Sometimes heterogeneous across studies	Assists in an integrated interpretation of the case
CSI (Central Sensitization Inventory) [22]	Central sensitization	Can identify a more complex pain context	Strongly influenced by comorbidities	Relevant as a confounding factor and indicator of clinical complexity

## Data Availability

No new data were created or analyzed in this study. Data sharing is not applicable to this article.

## Supplementary Materials

The following supporting information can be downloaded at: https://www.mdpi.com/article/10.3390/medicina62050999/s1, Table S1. Database-specific search strategy used for the conceptual narrative review.

## Abbreviations

The following abbreviations are used in this manuscript:

TMD	Temporomandibular disorders
DC/TMD	Diagnostic Criteria for Temporomandibular Disorders
RDC/TMD	Research Diagnostic Criteria for Temporomandibular Disorders
OBC	Oral Behavior Checklist
OBC–21	21 item Oral Behavior Checklist
GAD-7	Generalized Anxiety Disorder-7
PHQ-9	Patient Health Questionnaire-9
CSI	Central Sensitization Inventory
JFLS	Jaw Functional Limitation Scale
STAB	Standardized Tool for the Assessment of Bruxism
GCPS	Graded Chronic Pain Scale
