# Supplementary material for: Oral Behaviors, Anxiety, and Depression in Temporomandibular Disorders: A Conceptual Narrative Review Within the DC/TMD Axis II Framework
Source: Medicina (Kaunas). 2026 May 20;62(5):999. doi: 10.3390/medicina62050999 (PMC13208302; doi:10.3390/medicina62050999)
Supplement: Supplementary file 1 [file medicina-62-00999-s001.zip › medicina-4291961-supplementary.pdf]

Supplementary Material

Supplementary Table S1. Database-specific search strategy used for the conceptual narrative review

Database/source	Search block	Exact search string/query	Limits/filters	Date searched	Records retrieved/screened
PubMed/MEDLINE	DC/TMD, RDC/TMD, and Axis II	(("temporomandibular disorders"[MeSH Terms] OR "temporomandibular disorders"[Title/Abstract] OR TMD[Title/Abstract]) AND ("Diagnostic Criteria for Temporomandibular Disorders"[Title/Abstract] OR "DC/TMD"[Title/Abstract] OR "RDC/TMD"[Title/Abstract]) AND ("Axis II"[Title/Abstract] OR psychosocial[Title/Abstract] OR biobehavioral[Title/Abstract])) AND ("2001/01/01"[Date - Publication] : "2026/03/31"[Date - Publication]) AND english[Language]	English; Jan 2001-Mar 2026	March 2026	[245]
PubMed/MEDLINE	Oral behaviors and OBC	(("temporomandibular disorders"[MeSH Terms] OR "temporomandibular disorders"[Title/Abstract] OR TMD[Title/Abstract]) AND ("oral behavior"[Title/Abstract] OR "oral behaviors"[Title/Abstract] OR "oral behaviour"[Title/Abstract] OR "oral behaviours"[Title/Abstract] OR "Oral Behaviors Checklist"[Title/Abstract] OR "Oral Behaviours Checklist"[Title/Abstract] OR OBC[Title/Abstract] OR OBC-21[Title/Abstract] OR "awake bruxism"[Title/Abstract] OR parafunction*[Title/Abstract])) AND ("2001/01/01"[Date - Publication] : "2026/03/31"[Date - Publication]) AND english[Language]	English; Jan 2001-Mar 2026	March 2026	[455]
PubMed/MEDLINE	Anxiety, depression, and psychological distress	(("temporomandibular disorders"[MeSH Terms] OR "temporomandibular disorders"[Title/Abstract] OR TMD[Title/Abstract]) AND (anxiety[Title/Abstract] OR "anxiety symptoms"[Title/Abstract] OR "GAD-7"[Title/Abstract] OR depression[Title/Abstract] OR "depressive symptoms"[Title/Abstract] OR "PHQ-9"[Title/Abstract] OR "psychological distress"[Title/Abstract])) AND ("2001/01/01"[Date - Publication] : "2026/03/31"[Date - Publication]) AND english[Language]	English; Jan 2001-Mar 2026	March 2026	[1009]

Database/source	Search block	Exact search string/query	Limits/filters	Date searched	Records retrieved/screened
PubMed/MEDLINE	Oral behaviors and psychological symptoms	(("temporomandibular disorders"[MeSH Terms] OR "temporomandibular disorders"[Title/Abstract] OR TMD[Title/Abstract]) AND ("oral behavior"[Title/Abstract] OR "oral behaviors"[Title/Abstract] OR "oral behaviour"[Title/Abstract] OR "oral behaviours"[Title/Abstract] OR "Oral Behaviors Checklist"[Title/Abstract] OR "Oral Behaviours Checklist"[Title/Abstract] OR OBC[Title/Abstract] OR OBC-21[Title/Abstract] OR "awake bruxism"[Title/Abstract] OR parafunction*[Title/Abstract]) AND (anxiety[Title/Abstract] OR "GAD-7"[Title/Abstract] OR depression[Title/Abstract] OR "PHQ-9"[Title/Abstract] OR "psychological distress"[Title/Abstract])) AND ("2001/01/01"[Date - Publication] : "2026/03/31"[Date - Publication]) AND english[Language]	English; Jan 2001-Mar 2026	March 2026	[139]
PubMed/MEDLINE	Pain-related disability and jaw function	(("temporomandibular disorders"[MeSH Terms] OR "temporomandibular disorders"[Title/Abstract] OR TMD[Title/Abstract]) AND ("jaw functional limitation"[Title/Abstract] OR JFLS[Title/Abstract] OR "Jaw Functional Limitation Scale"[Title/Abstract] OR "pain-related disability"[Title/Abstract] OR disability[Title/Abstract] OR "Graded Chronic Pain Scale"[Title/Abstract] OR GCPS[Title/Abstract])) AND ("2001/01/01"[Date - Publication] : "2026/03/31"[Date - Publication]) AND english[Language]	English; Jan 2001-Mar 2026	March 2026	[584]
Scopus	DC/TMD, RDC/TMD, and Axis II	TITLE-ABS-KEY(("temporomandibular disorder" OR "temporomandibular disorders") AND ("Diagnostic Criteria for Temporomandibular Disorders" OR "DC TMD" OR "RDC TMD" OR "DC/TMD" OR "RDC/TMD") AND ("Axis II" OR psychosocial OR biobehavioral OR biopsychosocial)) AND PUBYEAR > 2000 AND PUBYEAR < 2027AND (LIMIT-TO(LANGUAGE, "English"))	English; 2001-2026; March 2026 export used as upper date boundary	March 2026	[292]
Scopus	Oral behaviors and OBC	TITLE-ABS-KEY(("temporomandibular disorder" OR "temporomandibular disorders") AND ("oral behavior" OR "oral behaviors" OR "oral behaviour" OR "oral behaviours" OR "Oral Behaviors Checklist" OR "Oral Behaviours Checklist" OR OBC OR "OBC-21" OR "awake bruxism" OR parafunction*)) AND PUBYEAR > 2000 AND PUBYEAR < 2027 AND (LIMIT-TO(LANGUAGE, "English"))	English; 2001-2026; March 2026 export used as upper date boundary	March 2026	[511]

Database/source	Search block	Exact search string/query	Limits/filters	Date searched	Records retrieved/screened
Scopus	Anxiety, depression, and psychological distress	TITLE-ABS-KEY(("temporomandibular disorder" OR "temporomandibular disorders") AND (anxiety OR "anxiety symptoms" OR "GAD-7" OR depression OR "depressive symptoms" OR "PHQ-9" OR "psychological distress")) AND PUBYEAR > 2000 AND PUBYEAR < 2027 AND (LIMIT-TO(LANGUAGE, "English"))	English; 2001-2026; March 2026 export used as upper date boundary	March 2026	[1133]
Scopus	Oral behaviors and psychological symptoms	TITLE-ABS-KEY(("temporomandibular disorder" OR "temporomandibular disorders") AND ("oral behavior" OR "oral behaviors" OR "oral behaviour" OR "oral behaviours" OR "Oral Behaviors Checklist" OR "Oral Behaviours Checklist" OR OBC OR "OBC-21" OR "awake bruxism" OR parafunction*) AND (anxiety OR "GAD-7" OR depression OR "PHQ-9" OR "psychological distress")) AND PUBYEAR > 2000 AND PUBYEAR < 2027 AND (LIMIT-TO(LANGUAGE, "English"))	English; 2001-2026; March 2026 export used as upper date boundary	March 2026	[156]
Scopus	Pain-related disability and jaw function	TITLE-ABS-KEY(("temporomandibular disorder" OR "temporomandibular disorders") AND ("jaw functional limitation" OR JFLS OR "Jaw Functional Limitation Scale" OR "pain-related disability" OR "Graded Chronic Pain Scale" OR GCPS OR "pain intensity" OR "functional limitation" OR disability)) AND PUBYEAR > 2000 AND PUBYEAR < 2027 AND (LIMIT-TO(LANGUAGE, "English"))	English; 2001-2026; March 2026 export used as upper date boundary	March 2026	[1371]
Google Scholar	Supplementary search: DC/TMD Axis II	"DC/TMD" "Axis II" "temporomandibular disorders"	First 100 relevance-ranked results screened; English-language relevance; 2001–Mar 2026 where identifiable	March 2026	100 screened
Google Scholar	Supplementary search: OBC	"temporomandibular disorders" "Oral Behaviors Checklist"	First 100 relevance-ranked results screened; English-language relevance; 2001–Mar 2026 where identifiable	March 2026	100 screened
Google Scholar	Supplementary search: oral behaviors and anxiety	"temporomandibular disorders" "oral behaviors" anxiety	First 100 relevance-ranked results screened; English-language relevance; 2001–Mar 2026 where identifiable	March 2026	100 screened
Google Scholar	Supplementary search: oral behaviors and depression	"temporomandibular disorders" "oral behaviors" depression	First 100 relevance-ranked results screened; English-language relevance; 2001–Mar 2026 where identifiable	March 2026	100 screened
Google Scholar	Supplementary search: GAD-7 and PHQ-9	"temporomandibular disorders" "GAD-7" "PHQ-9"	First 100 relevance-ranked results screened; English-language relevance; 2001–Mar 2026 where identifiable	March 2026	100 screened
Google Scholar	Supplementary search: jaw functional limitation	"temporomandibular disorders" "jaw functional limitation" "Axis II"	First 100 relevance-ranked results screened; English-language relevance; 2001–Mar 2026 where identifiable	March 2026	100 screened
